# Python workflow for the selection and identification of marker peptides—proof-of-principle study with heated milk

**DOI:** 10.1007/s00216-024-05286-w

**Published:** 2024-04-12

**Authors:** Gesine Kuhnen, Lisa-Carina Class, Svenja Badekow, Kim Lara Hanisch, Sascha Rohn, Jürgen Kuballa

**Affiliations:** 1 GALAB Laboratories GmbH, Am Schleusengraben 7, 21029 Hamburg, Germany; 2 Department of Food Chemistry and Analysis, Institute of Food Technology and Food Chemistry, Technical University Berlin, Gustav Meyer Allee 25, 13355 Berlin, Germany; 3 Hamburg School of Food Science, Institute of Food Chemistry, University of Hamburg, Grindelallee 117, 20146 Hamburg, Germany

**Keywords:** Python, Chemometrics, Proteomics, Feature identification, Processed milk, Mass spectrometry

## Abstract

The analysis of almost holistic food profiles has developed considerably over the last years. This has also led to larger amounts of data and the ability to obtain more information about health-beneficial and adverse constituents in food than ever before. Especially in the field of proteomics, software is used for evaluation, and these do not provide specific approaches for unique monitoring questions. An additional and more comprehensive way of evaluation can be done with the programming language Python. It offers broad possibilities by a large ecosystem for mass spectrometric data analysis, but needs to be tailored for specific sets of features, the research questions behind. It also offers the applicability of various machine-learning approaches. The aim of the present study was to develop an algorithm for selecting and identifying potential marker peptides from mass spectrometric data. The workflow is divided into three steps: (I) feature engineering, (II) chemometric data analysis, and (III) feature identification. The first step is the transformation of the mass spectrometric data into a structure, which enables the application of existing data analysis packages in Python. The second step is the data analysis for selecting single features. These features are further processed in the third step, which is the feature identification. The data used exemplarily in this proof-of-principle approach was from a study on the influence of a heat treatment on the milk proteome/peptidome.

**Graphical abstract:**

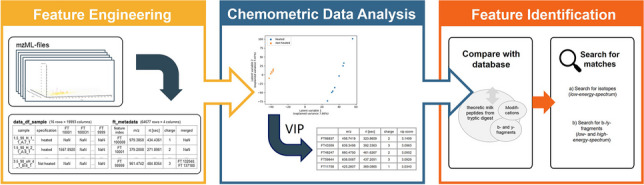

**Supplementary Information:**

The online version contains supplementary material available at 10.1007/s00216-024-05286-w.

## Introduction

Still, the most common way in food analysis is the processing of data (i.e., identification and quantification) with software provided by the manufacturer of analytical instruments/devices. However, this limits the possibilities and the potential gain of a study. Additionally, time-consuming building of “own” individual databases is necessary and the application of chemometric tools is not always possible. New evaluation algorithms cannot easily be integrated into the data processing, which also restricts the development of new approaches.

In general, data processing must deal with the steadily growing amount of data generated by analytical devices. There is a need to effectively process a large amount of data to enable an added value of knowledge [1, 2]. In this context, so-called chemometrics and machine learning are becoming more and more relevant for a more effective use of data [3]. The term chemometrics summarizes mathematical and statistical methods for the evaluation of analytical and chemical measurements [4–6]. In the context of chemometrics, the integration of programming languages is gaining popularity. These offer the customization of workflows and flexibility in the application of a broad range of evaluation tools and algorithms.

Python is an object-oriented programming language and distinguished by its quite simple syntax [7, 8]. It is already popular in the field of analytical chemistry and developed to a status, where it offers an extensive ecosystem for chemometric applications. Due to the availability of open-source packages, data processing is becoming much easier, and less programming knowledge is needed. Often the functions included in these packages are efficient and optimized for their application. Python itself is open source, which supports the exchange of information and the collaboration between researchers and comparing results from different, but closely related studies and datasets.

Especially in the field of mass spectrometry (MS), Python can enhance data evaluation, as MS produces large amounts of data and deals with diverse experimental approaches that need to be considered in the evaluation process. MS is used for a broad range of analytical targets with several different analytical techniques and devices. The complex datasets and influence factors can be combined by utilizing the Python ecosystem, being strongly supported by different Python software packages. They include valuable functions like the raw data import and the preprocessing of data. Python packages that have to be mentioned in the field of MS are *pyOpenMS* [9, 10], *pymzml* [11, 12], *Pyteomics* [13], and *AlphaPept* [14]. These offer diverse functions for general data processing. Beyond, there are also packages for more specialized devices or analytes, e.g., *gc-ims-tools* for GC-IMS data [15] or *GlyXtoolMS* for glycopeptide MS data [16]. Packages for machine learning (e.g., *Scikit-Learn* [17]) and deep learning (e.g., *TensorFlow* [18]) offer easy application of established data analysis tools, but also bear the potential of easily implementing new developments in bioinformatics. An essential part of the programming process is to bring the mass spectrometric data together with these available data science packages. More precisely, the data must be transformed to fit the data science packages. In the present study, this combination is called feature engineering. Once the data is transformed to the specific need, diverse statistical and chemometric analyses can be performed. In addition, Python offers established packages for data visualization (e.g., matplotlib [19]).

For the evaluation of MS data from proteomics bottom-up experiments, the identification of potential marker peptides is the challenge. Therefore, the specific proteolysis enzyme (in most cases trypsin), the b- and y-fragmentation, and posttranslational modifications (PTM) of the peptides need to be considered [20]. The identification of peptides is to date rarely integrated in Python-based algorithms. Researchers often utilize other software for example Mascot [21] or Sequest [22] rather than using Python for the identification of the peptides. In the traditional software approaches, the modifications are limited to the software’s database, and case-specific peptide modifications (e.g., Maillard reaction products) might not be taken into account. With an individual database that can be built in Python and used for the identification process, experiment-specific modifications could be considered as well.

The aim of the present study was to develop a proof-of-principle workflow, bringing feature engineering, chemometric data analysis, and the identification of unique peptides together. The further outcome of such an algorithm can be the identification and selection of potential peptides, which can be used as marker compounds for a certain research question. There were already studies dealing with the evaluation of mass spectrometric and proteomic studies with Python. Most of them focused on the bioinformatic approach, offering packages with general functions for evaluation [9, 11, 23–25]. However, these were mainly utilized for initial data handling, but often missed the follow-up integration of machine learning and chemometric approaches.

From a literature and a database survey, it seems that there is actually no study presenting a Python workflow for the selection and identification of marker peptides from (experimental) mass spectrometric data. In turn, there are studies applying Python in single steps of the data evaluation rather than a whole workflow [26–28]. For example, Fiedler et al. used Python to compare peptides with a database of immunogenic sequences [26]. Solazzo et al. used Python to perform an in silico tryptic digest of proteins and the determination of potential permutations of these peptides [27]. Chen et al. incorporated Python in the step of mapping protein and genome data [28]. These three examples demonstrate how Python can already improve studies by incorporating it in single steps. The development of a continuous workflow aimed at herein proceeds all steps of the evaluation in the Python ecosystem. By integrating the programming language into the whole process of data evaluation, such a continuous workflow is supposed to become more traceable and customized**.** The workflow implements functions from existing Python packages, which are established and simplify the data analysis. For not being too conceptual, this approach was developed at hand of heat-induced changes in milk (proteins), as a model. The experimental peptide data was acquired after tryptic hydrolysis by UPLC-IMS-qTof. It was aimed at a rather simple but effective experimental design that offers a future basis for the development of similar workflows in food chemistry and analysis.

## Materials and methods

### Reagents

Water and acetonitrile (both HPLC-grade) were obtained from VWR International GmbH (Darmstadt, Germany). Formic acid was purchased from Biosolve B.V. (Valkenswaard, Netherlands). α-Lactalbumin was purchased from US Biological Inc. (Salem, MA, USA). β-Lactoglobulin, BSA (bovine serum albumin), α-casein, β-casein, and κ-casein were purchased from Sigma-Aldrich Chemie GmbH (Schnelldorf, Germany), as well as acetic acid, dithiothreitol (DTT), iodoacetamide (IAA), urea, and sodium bicarbonate. Ammoniumhydrogencarbonate was purchased from Thermo Fisher Scientific Inc. (Waltham, MA, USA). The enzyme trypsin (from porcine pancreas, specific activity: 5000 usp-u/mg protein) was purchased from Carl Roth GmbH & Co. KG (Karlsruhe, Germany). The calibration standard and the lock mass (leucine enkephalin) were purchased from Waters Corp. (Milford, MA, USA).

### UPLC-IMS-qTof dataset

Two different types of milk were used for the study, only differing in the fat content. One batch of UHT (ultrahigh-temperature processing) milk with 1.5% fat and one batch of UHT milk with 3.5% fat from the organic brand *Gläserne Molkerei GmbH* (Dechow, Germany). From both batches, eight samples were taken each, leading to 16 samples in total. Half of the samples, with an equal share of the two batches, were heated to 90 °C for 10 min and then cooled down to room temperature. Samples were diluted 1:100 with water.

Standard milk proteins (αs1-casein, αs2-casein, β-casein, κ-casein, α-lactalbumin, β-lactoglobulin, BSA) were used as solutions (caseins: 4 mg/mL in sodium bicarbonate; α-lactalbumin, β-lactoglobulin, BSA 4 mg/mL in water). Four samples of each protein were used in total. Two of each protein samples were heated to 90 °C for 10 min like the milk samples. The other two samples were not heated.

Samples were digested with trypsin according to a protocol presented by Giansanti et al. [29]. Briefly, the milk samples and the standard protein solutions were concentrated until dryness and redissolved in 2 M aqueous urea. The digestion was executed with an incubation time of 12 h at 37 °C. The digestion was followed by solid-phase extraction (SPE) with Sep-Pak® C18 cartridges (Waters GmbH, Eschborn, Germany) [29]. Afterwards, the peptide solutions were concentrated to dryness and redissolved in 500 µL 0.1% formic acid in water.

Mass spectrometric analysis was performed with an Acquity I-Class UPLC (ultrahigh performance liquid chromatography) system coupled with a Vion IMS-QToF-MS (ion mobility spectroscopy quadrupole-time-of-flight mass spectrometer) (all by Waters Corp., Milford, MA, USA). For the separation with UPLC, an ACQUITY® UPLC BEH C8 column was used (150 mm × 2.1 mm, 1.7 µm, 130 Å; Waters Corp., Milford, MA, USA). The column temperature was set to 40 °C. The used flow was 0.2 mL/min. As mobile phase A water with 0.1% formic acid was used. Mobile phase B was acetonitrile with 0.1% formic acid. The following gradient was used: 0.0 min (99% A), 1.0 min (99% A), 10.0 min (58% A), 12.0 min (15% A), 15.0 min (15% A), 16.5 min (99% A), 19.5 min (99% A). Two microliters was injected. The autosampler was set to 10 °C. For the mass spectrometric detection, positive ion mode was used. Parameters were set as follows: source temperature, 120 °C; desolvation temperature, 450 °C; cone gas flow, 50 L/h (nitrogen); desolvation gas flow, 800 L/h (nitrogen); capillary voltage, 0.50 kV; sample cone voltage, 40 V; source offset voltage, 80 V. The used mass spectrometric device was an MS^E^ instrument, producing simultaneously *low*- and *high-energy-spectra*. The *low-energy-spectra* were obtained using 4 eV as collision energy and the *high-energy-spectra* were obtained by using a ramp with elevated collision energy starting at 15 eV and ending at 45 eV. The mass range was set to mass-to-charge-ratio (*m/z*) 50–2000.

### Computational framework

Data analysis and statistics were performed with a notebook (Processor: Intel(R) Core(TM) i5-10210U; CPU: 1.60 GHz, 2.11 GHz; RAM: 16 GB). The programming language Python (Version 3.9.16) [30] and the development environments JupyterNotebook (Version 6.5.3) and PyCharm Community (Version 2020.3.3) were used for the analysis. For the data processing, the packages used are listed in Table 1.

**Table 1:** Summary of the Python packages that were used in the presented study for the data processing. Packages listed with version and short description

Package	Version	Description
*pyopenms*	2.7.0	Python bindings for the OpenMS library; library for mass spectrometry, proteomics, and metabolomics
*pandas*	1.5.3	Data structures for data analysis
*scikit-learn*	1.2.2	Modules for machine learning and data mining
*numpy*	1.24.2	Scientific array computing
*matplotlib*	3.7.2	Data plotting and visualization
*seaborn*	0.12.2	Statistical data visualization

The acquired data was converted from the producer-specific uep-format to the universal mzML-format with MSConvert (ProteoWizard, Version 3.0.20340) [31]. In the process of the data conversion, ion mobility was compressed to reduce the data size and the needed computational compacity.

### Data processing and analysis

The manual settings are specified for the functions, which were used from already publicly available packages. Unless specified otherwise, the default setting was used. For reading mzML-files (*MzMLFile*) and feature finding (*FeatureFinderCentroided)*, functions from *pyOpenMS* were used. The *FinderFinderCentroided* settings were not changed manually. The standard settings can be found in the online *OpenMS* documentation [32]. In the second process, data was centroided. Afterwards, data was transformed into *pandas.DataFrames*. Partial least square discriminant analysis (PLS-DA) was performed using *scikit-learn*. The number of components was set to two. The variable importance in the projection (VIP) score was calculated to select features with the biggest influence on the PLS-DA model. The VIP score calculation was proceeded as presented in the *gc-ims-tools* package [15]. For the identification of features, the protein sequence from different milk proteins was read as fasta-files, which were loaded from uniprot.org (accessed 15.08.2023). The proteins used as models were as follows: αs1-casein, αs2-casein, β-casein, κ-casein, α-lactalbumin, β-lactoglobulin, and BSA. For their theoretic tryptic digestion, the *ProteaseDigestion* from *pyOpenMS* was used. Missed cleavage was set thereby to two. The *TheoreticalSpectrumGenerator* from *pyOpenMS* was used for calculating the b- and y-fragments of peptides (“add_b_ions,” “add_a_ions,” “add_losses,” “add_metainfo” were all set to True). The *FineIsotopePatternGenerator* from *pyOpenMS* was used for calculating the isotope pattern of peptides and modified peptides. The cut-off for the *FineIsotopeGenerator* was set to 0.05%, which means that isotopes covering 99.95% of the abundance are returned by the function. Through the study, plots were generated with *matplotlib* and *seaborn.*

## Results

### Feature engineering

**Fig. 1:**
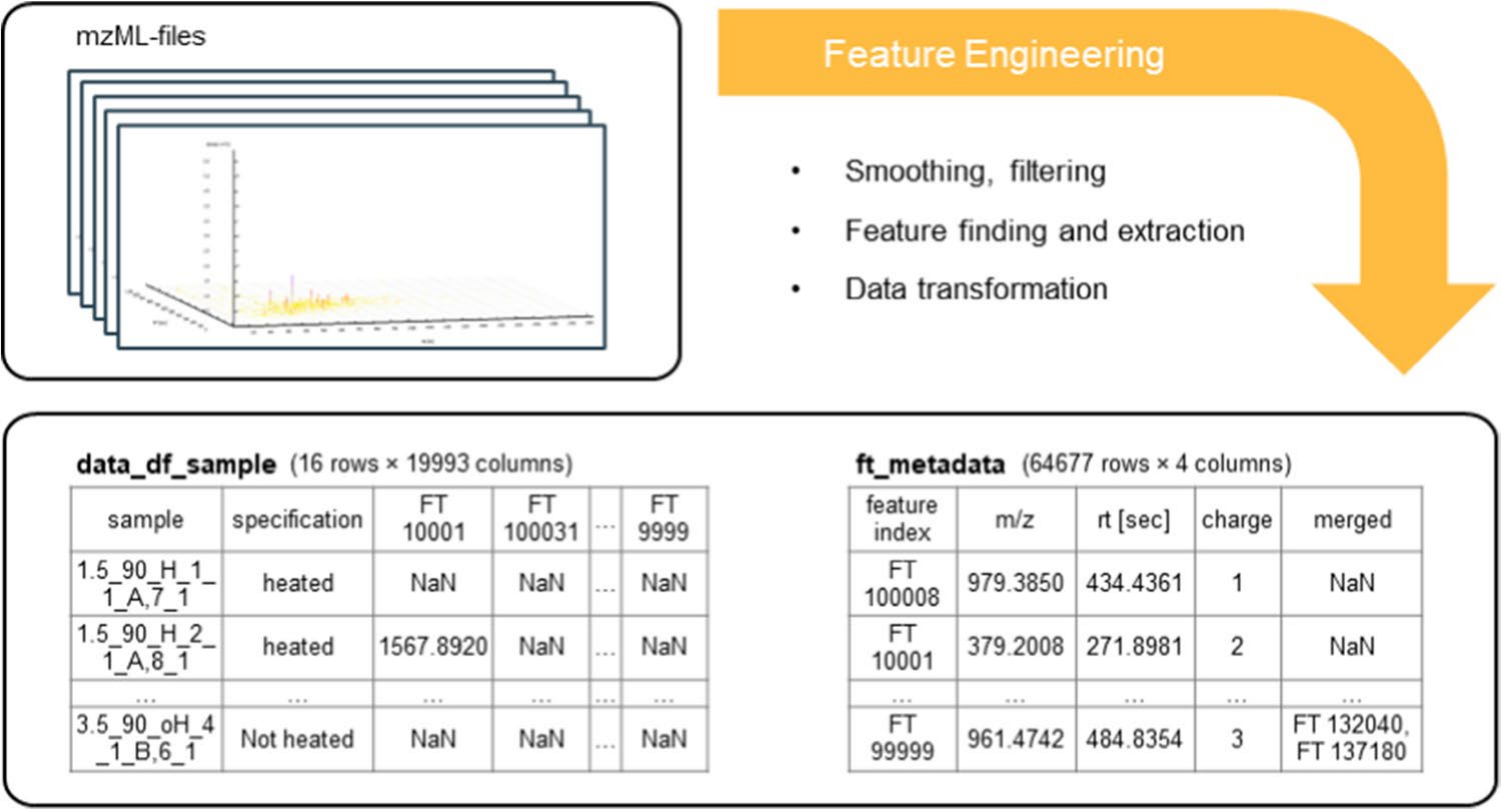
Visualization of the feature engineering process. Shown is the transformation of the mass spectrometric data (mzML-files) to two *pandas.DataFrames*. The data frame on the left (“data_df_sample”) contains the intensities of the features in each sample. The data frame on the right (“ft_metadata”) contains the metadata which defines each feature. The transformation of the data into this shape was proceeded to enable the application of data analysis tools. In the process of feature engineering smoothing of the data, feature finding, feature extraction, and data transformation were proceeded. NaN means “Not a Number” and is used when no data is available

The goal of feature engineering was to extract features and to prepare the data for the application of chemometric data analysis. *pyOpenMS* [9, 10] functions were used throughout the data processing. Among other applications, it was used to read the data in the mzML-format. For the extraction of features, *FeatureFinderCentroided* (*pyOpenMS*) was used. The extracted features were saved in two data frames. A *pandas.DataFrame* is a data structure provided by the Python package *pandas*, which is comparable to tables [33]. One of the data frames contained the intensities of the features in the samples. The other data frame defines the features by containing the *m/z*, the retention time in seconds, and the estimated charge ( +). The charge was estimated by the *FeatureFinderCentroided* based on the isotopic pattern of the feature [34]. In the following, the size of the data frames was reduced by finding features that were similar and merging these into one feature. This merging was performed with an absolute tolerance in the *m/z* of 0.01 and an absolute tolerance in the retention time of 5 s. A specification was inserted for labelling, when a dataset was from a sample that was treated with heat, not treated with heat, or when it was a pure milk protein. The samples (heat *vs.* non-heated) were selected and saved in a separate data frame. Till then, the feature engineering was performed with data from samples and the pure milk proteins. The data from these standard proteins were used to facilitate feature identification later on, but not used for the chemometric analysis. Figure 1 shows the resulting shape of the data as the two extracted data frames. A more detailed presentation of the feature engineering is shown in Fig. S1. After feature engineering, 19,991 features were extracted and used for the statistical analysis. Different chemometric tools for example principal component analysis (PCA) or partial least square regression (PLSR) can be applied on the data at this stage according to the design and the aim of the study.

### Chemometric data analysis

**Fig. 2:**
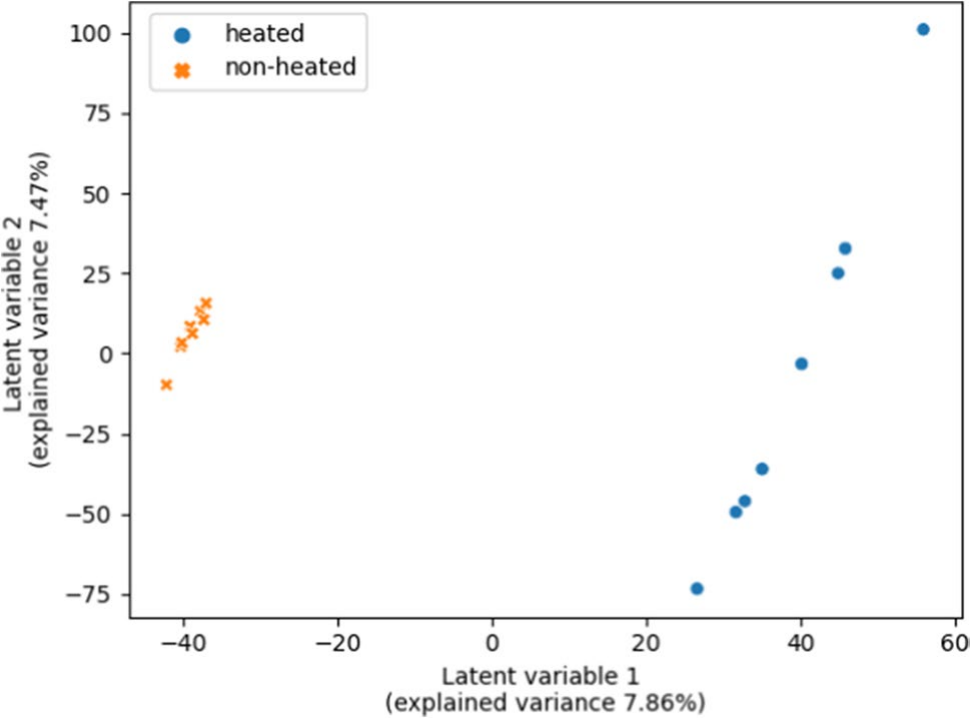
PLS-DA plot of the milk samples. Orange spots (x-shaped) show the samples that were non-heated in the sample preparation. Blue spots are the samples which were heated in the sample preparation. The plot was generated with matplotlib and seaborn

PLS-DA is a chemometric tool for classification. It is a supervised method and therefore needs labelled data. In this case, PLS-DA was the preferred classification method due to its performance on datasets with a large number of variables and a limited number of samples [35, 36]. It was also chosen for its usability for feature selection [37]. By performing the PLS-DA, a set of latent variables is modelled to discriminate the data between the given categories [38, 39]. In the present study, the categories were “heated” and “non-heated.” The “heated” samples are UHT milk samples that were treated with heat at sample preparation. The “non-heated” samples are UHT milk samples that were not treated with heat at sample preparation. Figure 2 shows the PLS-DA plot of the data.

**Fig. 3:**
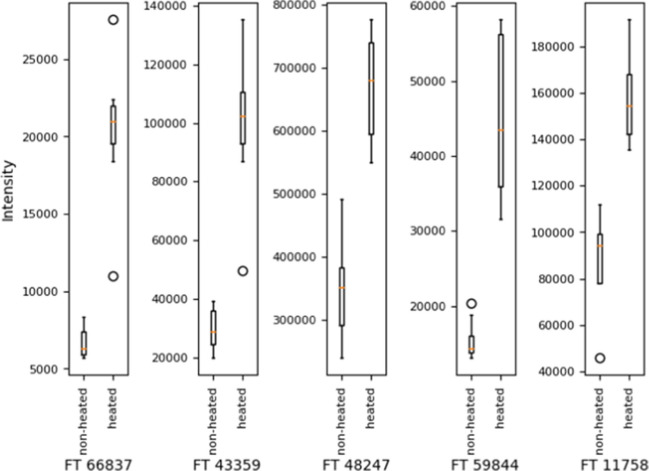
Boxplot of the intensities of the five top-scoring features in the samples. For each feature, the intensities in the non-heated and the heated samples are presented as boxplots. The circles represent outliers. The plot was generated with matplotlib

For selecting specific features, the variable importance in projection (VIP) for the features was calculated and the five features with the highest VIP scores were selected. The VIP score is a method for determining the influence of a feature to the PLS [15, 40]. The stronger the influence on the discrimination, the higher the VIP score. As a guideline, VIP scores > 1 are considered significant, but an individual decision is advisable [15, 40]. In this case, the five features with the highest VIP scores were selected. Table 2 shows these features with their defining data and VIP scores. Figure 3 shows the graphical visualization of the five selected features as boxplots.

**Table 2:** Top five features selected for the discrimination of heated and non-heated milk samples

Feature name	*m/z*	rt [s]	Charge ( +)	VIP score
FT66837	458.7419	323.8609	2	3.1409
FT43359	639.3498	392.3363	3	3.0963
FT48247	880.4750	461.6267	2	3.0952
FT59844	638.0087	437.2051	3	3.0929
FT11758	425.2607	369.0865	1	3.0340

**Fig. 4:**
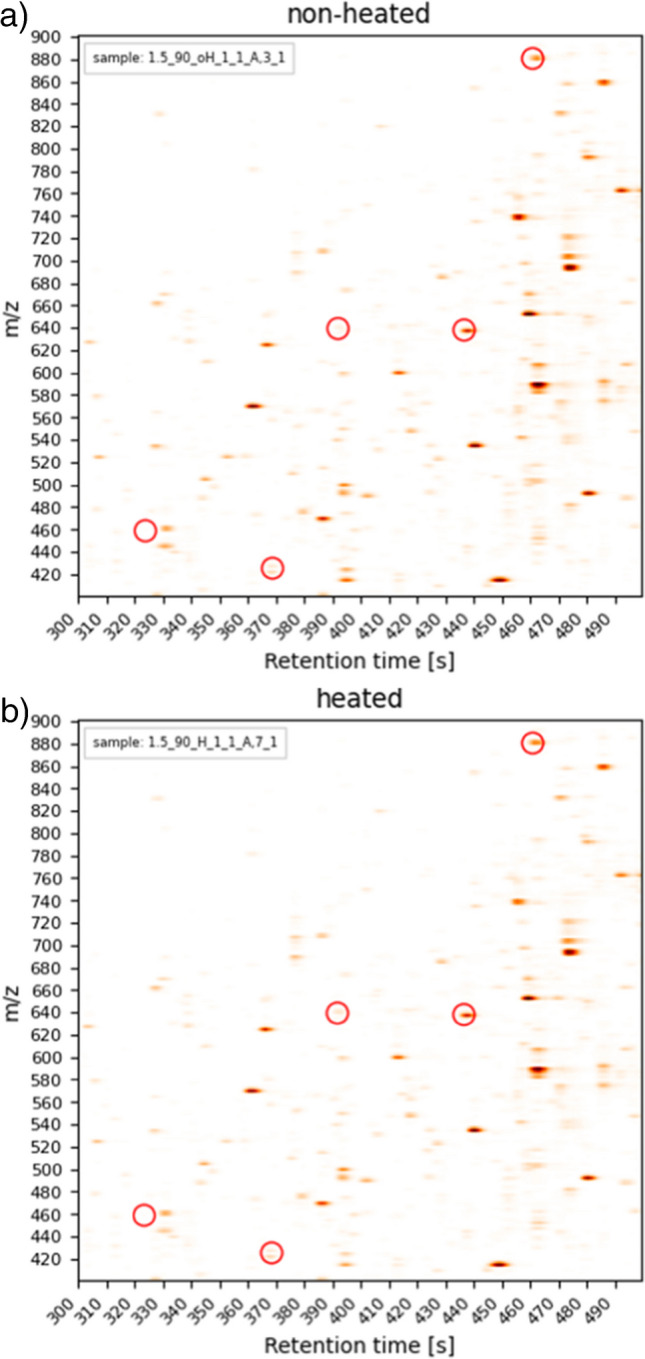
2D plot of the selected features in the MS spectra. Spectra were filtered by a retention time and *m/z* window based on the values of the features. **a** The spectrum shows a sample without further heat treatment. **b** The spectrum shows a sample with heat treatment. Both samples UHT milk with 1.5% fat. Top-scoring features are marked with red circles. The plots were generated with matplotlib

The selected features were in a *m/z* range from 425 to 880 and a retention time range from 323 to 461 s (5.40 min and 7.69 min). Figure 4 shows a 2D plot of a cutout of the spectra with the selected features marked. Figures 3 and 4 show that the selected features appeared in heated and non-heated samples and only differ in intensity, all of them with increased intensities by thermal treatment. Moreover, the dataset was filtered for features that only appeared either in the heated or in the non-heated samples, but none were found. The fact that all features were found in both datasets (heated and non-heated) could be explained by the pasteurization step in the factory. All milk samples were from one single batch of UHT milk, which means that the milk is pasteurized with heat in the factory. The short heat treatment due to pasteurization can already lead to the formation of these heat-induced features, but in lower concentrations, as the initial treatment in the factory was not as intense as the treatment done additionally in the present study. The concentrations are increased by the heat treatment of the milk at 90 °C.

### Feature identification

**Fig. 5:**
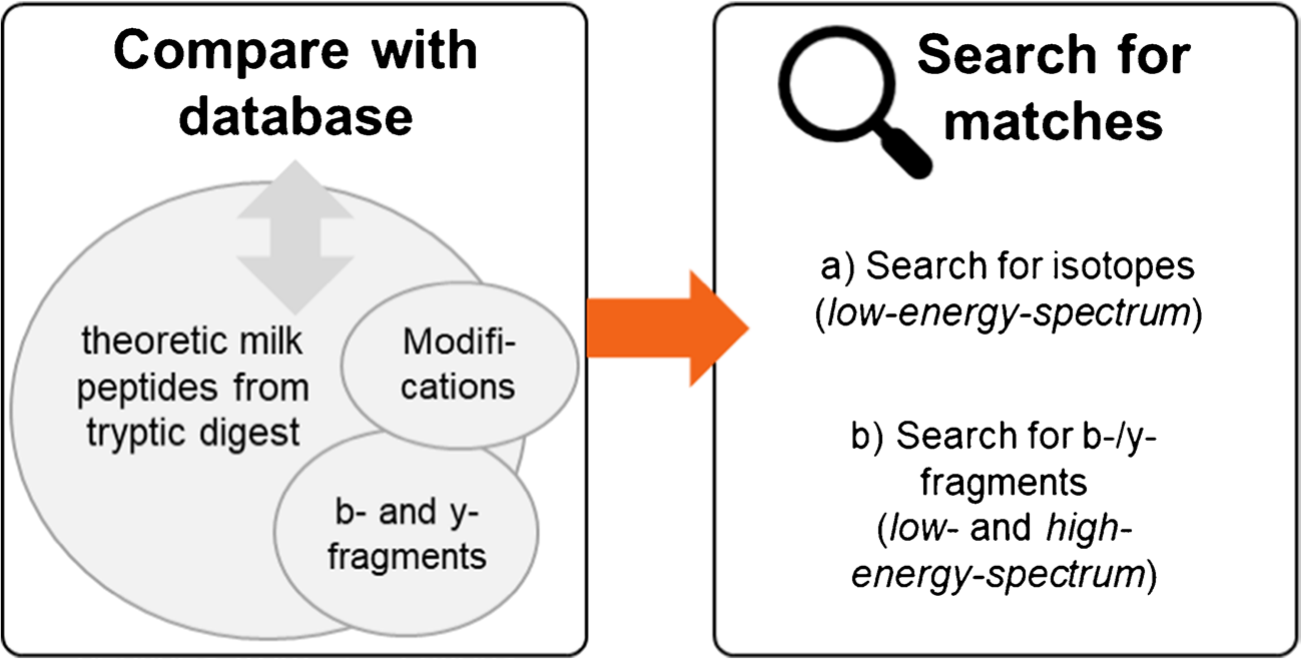
Schematic workflow of the feature identification. The process is split into two parts. Firstly, potential matches were searched. Therefore, the *m/z* of a feature was compared to theoretic *m/z* of peptides resulting from tryptic digest of milk proteins, as well as fragments and modifications of these peptides. In the second step, the spectra of the features are searched for isotopes and fragments of the potential matches

Figure 5 shows the schematic workflow for the identification of selected features. The feature identification is split into two main parts. In the first part, the features were compared to peptides that can theoretically be produced by the tryptic digestion of milk proteins.

The pure milk proteins for the comparison were αs1-casein, αs2-casein, β-casein, κ-casein, α-lactalbumin, β-lactoglobulin, and BSA. The amino acid sequences and the *m/z* of the tryptic peptides were calculated with the *ProteaseDigestion* function from *pyOpenMS*. Two missed cleavages were tolerated. Modifications of the peptides were considered as mass shift. Based on the literature on reactions in the heat treatment of milk [41–46], Maillard products like lactulosyllysine and oxidation products were considered. Besides, methylation and acetylation were considered as well. Furthermore, the b- and y-fragments of each peptide were calculated with the *TheoreticalSpectrumGenerator* function from *pyOpenMS*. The tolerance for the comparison of a feature and the peptides (modified, unmodified, fragmented, non-fragmented) was 0.001%. The tolerance between the observed *m/z* and theoretical *m/z* values is normally given as ppm. As it is used here as a parameter for a mathematical calculation process that was used flexibly for different *m/z*, a relative tolerance was preferred.

In the second part of the identification process, the potential matches were investigated further regarding isotopes and fragments. For potential matches, the *low-energy-spectra* were analyzed in order to find isotope signatures of a peptide. Therefore, the isotopes of a peptide (with or without a modification) were calculated with the *FineIsotopePatternGenerator* from *pyOpenMS*. These calculated masses of the isotopes were compared to the detected peaks in a spectrum at a defined time. The comparison was performed with a tolerance of 0.001%. The *low-* and *high-energy-spectra* were investigated, when b- and y-fragments of the peptide were detected. The b- and y-fragments of the peptides were calculated with the *TheoreticalSpectrumGenerator* from *pyOpenMS*. The received masses of the fragments were compared to the detected peaks in a spectrum at a defined time. This comparison was also performed with a tolerance of 0.001%.

By investigating the spectra for isotopes and fragments of the potential matches, it was determined if a potential match has an acceptable fit for the feature. It was considered if isotopes and if a variety of fragments were found. When using this workflow for the identification, three of the five selected features were matched with modified, unmodified, or fragments of the pure milk peptides. The results are presented in Table 3. In order to verify the results, the acquired data of the standards were searched for the features for gaining more information. In the case of these five identified features, no further information could be obtained, as the features could not be identified in the acquired data of the standard protein. The influence of the matrix on changes in the proteome is already described in the literature [47–49].

**Table 3:** Selected features with the results of the identification. The selected and detected features are listed with the feature name, *m/z*, charge, and the matched peptide with protein origin and if applicable fragment type or modification. beta_casein = β-casein, as1_casein = αs1-casein, beta_LG = β-lactoglobulin

Observed features			Database match		
Feature name	*m/z*	Charge ( +)	Peptide	Protein	Fragment/modification
FT66837	458.7419	2	No match found in the limitations of the search parameters		
FT43359	639.3498	3	VLPVPQKAVPYPQR	beta_casein	Lactulosyllysine
FT48247	880.4750	2	HQGLPQEVLNENLLR	as1_casein	-
FT59844	638.0087	3	No match found in the limitations of the search parameters		
FT11758	425.2607	1	ALPMHIR	beta_LG	y3 +

**Fig. 6:**
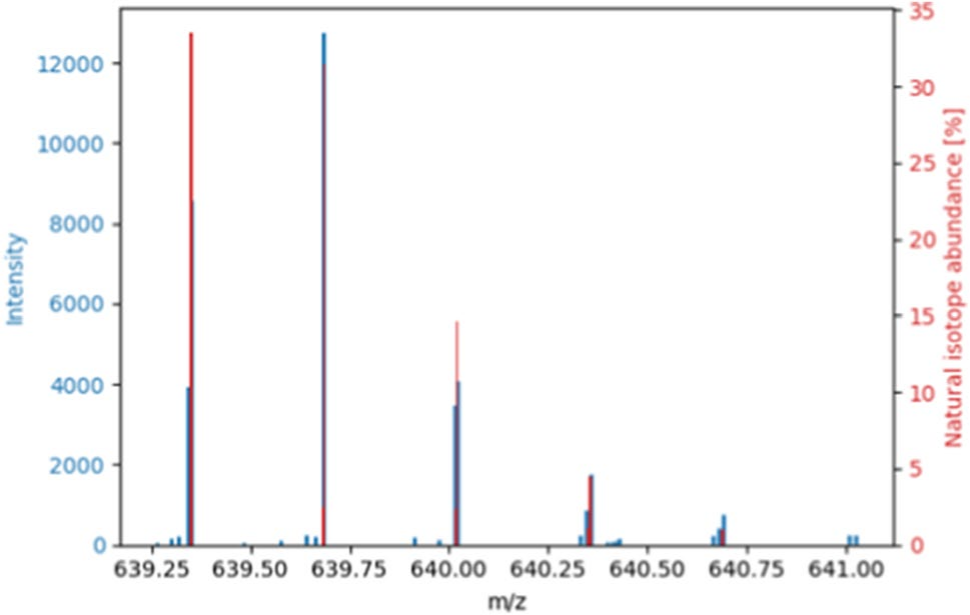
Cut-out of the *low-energy-*spectrum from a heated milk sample. Shown are the isotopes of the feature FT43359 as blue signals. The red signals show the calculated isotopes for the peptide VLPVPQKAVPYPQR modified with lactulosyllysine. The spectrum has a retention time of 392.3320 s. The assigned peaks are listed in Table 4. The plot was generated with matplotlib

**Fig. 7:**
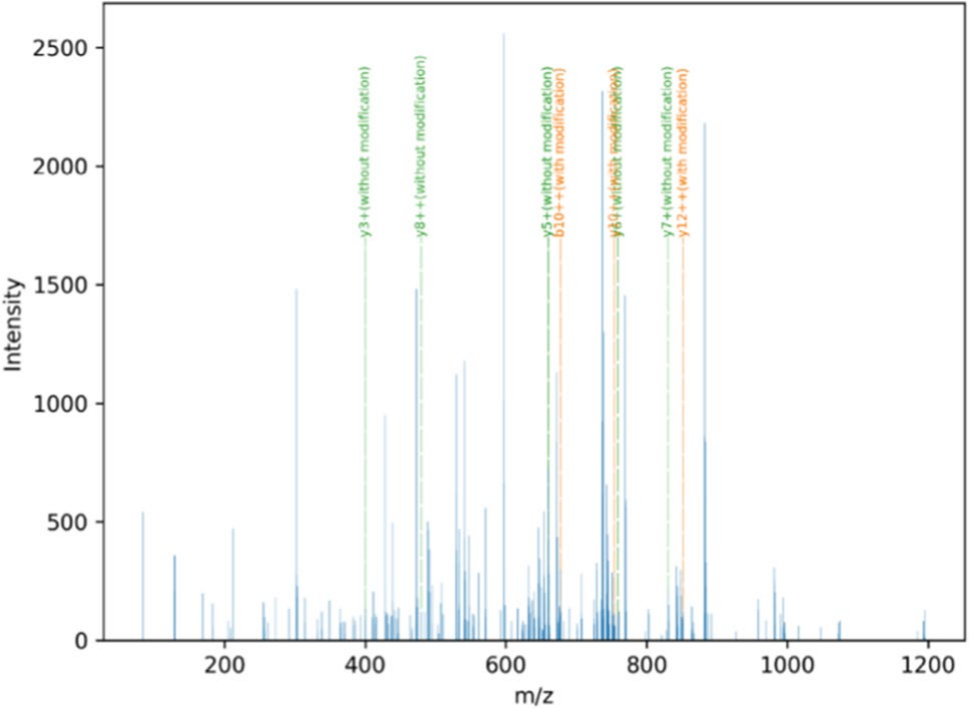
*High-energy-*spectrum of heated milk sample at the retention time of 392.5311 s. At the selected retention time, the feature FT43359 was detected in the *low-energy-*spectrum. The observed signals are displayed in blue. The theoretic b- and y-fragments that were associated by the approach are marked in orange (with the modification) and in green (with the modification). All fragments acquired at the retention time are shown in blue. The plot was generated with matplotlib

One feature (FT43359) was assigned to a peptide with lactulosyllysine as a modification. Figure S2 shows the output of the identification for this feature. Lactulosyllysine is a common modification of milk proteins caused by heat [44, 50, 51]. It is an Amadori product of the lysine side chain and lactose. Due to the glycation of lysine, there is no cleavage site for trypsin anymore, which normally cleaves the peptide bond after lysine [46, 52]. The assigned peptide was VLPVPQKAVPYPQR (position in intact β-casein: 185–198, MC: 1), which has a lysine in the middle. Isotopes of the peptide were found with charges 2 + and 3 + . The isotopes with the charge of 3 + are shown in Fig. 6. A comparison of the detected peaks and the assigned isotopes are listed in Table 4. Smaller y- and b-fragments were found without the modification as well as larger fragments with the modification, which supports the modification located in the middle. The comparison of the detected peaks in the *high-energy*-spectrum at the determined retention time and the assigned fragments are listed in Table 5. Figure 7 shows the corresponding *high-energy-*spectrum.

**Table 4:** Comparison of observed signals and assigned isotopes of the peptide VLPVPQKAVPYPQR modified with lactulosyllysine. The peaks are selected from the *low-energy*-spectrum (retention time: 392.3320 s) of a heated sample. The isotope *m/z* and the natural isotope abundance were calculated by the *FineIsotopePatternGenerator* from *pyOpenMS*. The comparison was made with a tolerance of 0.001%. Not assigned were the isotopes *m/z* 639.6836 (2.4727%), *m/z* 640.0193 (1.9260%), *m/z* 640.3538 (1.8123%), and *m/z* 640.6882 (0.8429%)

Observed signals		Theoretic isotopes of “VLPVPQKAVPYPQR”	
Observed *m/z*	Observed intensity	Isotope *m/z* (charge: 3)	Natural isotope abundance [%]
639.3521	8562.3193	639.3512	33.4725
639.6852	12,735.5996	639.6857	31.4965
640.0159	3473.6177	640.0180	2.3267
640.0223	4073.2292	640.0201	14.6483
640.3470	849.0919	640.3525	1.0821
640.3589	1751.6575	640.3546	4.4889
640.6931	741.7656	640.6891	1.0196

**Table 5:** Comparison of the signals which were detected in the *high-energy*-spectrum (retention time: 392.5311 s) of a heated milk sample. The signals were assigned to fragments of the peptide VLPVPQKAVPYPQR (unmodified or modified with lactulosyllysine). The detected peaks are listed with their *m/z* and intensity in the spectrum. The associated theoretic fragments are listed with the fragment type and with the *m/z*. Theoretic *m/z* were calculated by the *TheoreticalSpectrumGenerator* in *pyOpenMS*. They are listed as the *m/z* or the *m/z* with a mass shift due to the potential modification (lactulosyllysine). The tolerance in the *m/*z between the matched peak and the theoretic fragment was 0.001%

Observed signals		Theoretic fragments “VLPVPQKAVPYPQR”		
Observed *m/z*	Observed intensity	b-/y-fragment type	Fragment *m/z* (unmodified)	Fragment *m/z* (with the potential modifications)
400.2311	134.5270	[y3 +]	400.2303	
479.7739	120.0168	[y8 + +]	479.7771	
633.3450	180.2990	[y11-H3N1 + +]	633.3537	
651.3686	223.3610	[b13-H3N1 + +]	651.3663	
660.3440	728.9329	[y5 +]	660.3464	
660.3527	417.1433	[y5 +]	660.3464	
759.4187	101.1215	[y6 +]	759.4148	
830.4510	97.5177	[y7 +]	830.4519	
633.3182	59.2630	[y8-H3N1 + +]		633.3166
677.3728	97.1870	[b10 + +]		677.3792
677.3826	298.0854	[b10 + +]		677.3792
744.9073	256.0014	[a11 + +]		744.9134
744.9200	135.6328	[a11 + +]		744.9134
754.3870	112.0509	[y10 + +]		754.3856
831.4411	82.4058	[y12-C1H2N2 + +]		831.4353
843.9350	230.5972	[y12-H3N1 + +]		843.9329
852.4436	76.6360	[y12 + +]		852.4462
852.4546	122.7159	[y12 + +]		852.4462

The identification process does not calculate a score for the fit; it should be interpreted individually. The assigned fragments as presented in Table 5 should be interpreted in the individual context of the feature. The association is mathematical with the given tolerance. The y8 +  + -fragment was identified mathematically (Table 5). However, from a chemical-structural point of view, this fragment does not make any sense, because it misses the modification, which should be present in the y8-fragment. This modification is expected to be located at the lysine, the seventh amino acid counting from the *N*-terminus. This y8-fragment needs to undoubtedly include this lysine (Table 5).

The *high-energy*-spectra usually contain several fragments. It should be noted that these fragments can derive from different precursors [53]. Consequently, not all peaks can be assigned exclusively to one feature. Moreover, due to the large number of detected peaks, false assigning of peaks is more likely.

The outputs for the features FT48247 and FT11758 are shown in Fig. S3 and Fig. S4 (Supplementary Material). These features were evaluated in the same manner as the other features. They were assigned to peptides originating from αs1-casein and β-lactoglobulin.

## Discussion

In this study, a way of processing mass spectrometric data without device-specific software is shown. The workflow demonstrates a way of evaluating proteomic studies with the programming language Python. In general, most studies using Python for data analysis often offer package solutions for the evaluation. Röst et al. presented pyOpenMS as a package with diverse application possibilities in MS [9]. Another example is the study of Abdrakhimov et al. [23]. They presented the package Biosaur, a package for LC-IMS-MS [23]. These two are just examples; there are also various other packages that have more or less specific areas of application. Less often represented are studies that give explicit examples of the evaluation of an experiment, especially without promoting a single package.

The presented workflow shows the evaluation of a study on heat-induced changes in milk as a proof-of-principle. The workflow contains the evaluation steps feature engineering and chemometric data analysis as well as the (final) identification of peptides. The splitting into three main parts emphasizes the modular system of chemometric programming.

The presented algorithm of the feature engineering ends in a data structure that can be used for a wide range of chemometric tools offered by the Python environment. This structure is based on two data frames. One data frame contains the intensities of the features in the samples. The other data frame defines the features by the *m/z*, the retention time, and the charge. This structure is comparable to a data structure that was the basis for preprocessing of data presented by Riquelme et al. [54]. Their focus was on the preprocessing of mass spectrometric (small molecule) metabolomics data for quality evaluation and data curation. Riquelme et al. [54] introduced the package *TidyMS*. For the data analysis with TidyMS, they structured the data in a similar way with data frames as presented here. They stored the intensities of the features in one data frame and the data that describe the feature (like *m/z* and retention time) in another data frame. A third data frame was used to store information about the samples. Those authors further showed how such a transformation into a data frame-based structure offers a basis for different studies and different research questions [54].

In general, the use of PLS-DA for classification in chemometrics is well established [55]. In most studies, this approach is applied using a combination of different individual software. As an example, Núñez et al. [56] created feature data from (mass spectrometric) raw data with the software MZmine after data conversion with the software MSConvert. Those authors performed the chemometric analysis by utilizing the software Solo [57]. Their workflow demonstrates how different types of software are combined and used for data evaluation. In comparison to the present study, it becomes evident that the steps that are classified by the different types of software utilized by Núñez et al. [56] are comparable by the split into three main steps that was done in the present study, as well. The gathering of the steps in a Python workflow, as shown in the present study, enables an easy comparison between processed and raw data. Furthermore, a comparison of diverse *Machine Learning* tools can be achieved when the data is processed and evaluated in Python as Mendez et al. demonstrated [58].

PLS-DA was used in the present study for feature selection and not as a predictive model. For selecting a small number of features, overfitting is less relevant. An overfitted model is fitted too well to the calibration data and underperforms to predict new data [4]. For a prediction, the issue of overfitting can be minimized by further preprocessing, filtering of data, and/or cross-validation [59]. In the present study, PLS-DA was used in combination with the calculation of VIP scores. The identified features are potential marker peptides, representing the heat-induced change of the milk proteome. The useability of the combination of PLS-DA and VIP scores was already shown by Farrés et al. [40]. They compared VIP scores and selectivity ratio for feature selection from partial least square models. Both methods showed comparable results with only slightly different results in the three datasets. Christmann et al. utilized PLS-DA and VIP scores for feature selection in the preprocessing of data before different classification models [37].

The aim of the present study was the selection of marker peptides in a situation where the proteome of food is altered, but the significant threshold level for identifying it is not obvious. (Bio-)Markers are frequently used in food authenticity and food quality [49, 60–63]. Examples of using marker peptides are the differentiation of fish species [63], the determination of the content of whey protein in cheese [49], or peanut allergen detection in different foods [62]. The presented workflow shows a way to select potential markers and could be transferred to other research questions.

## Conclusion

The approach for the identification presented herein is a rather simple one. So-called features, representing potential unique compounds or fragments etc., can be identified with this approach. The simplicity of the approach makes it easy to adapt or adjust for other users. Here, the identification was based on isotopic and fragmentation patterns of milk peptides. Due to the MS^E^ fragmentation of the used mass spectrometer, fragments from diverse precursors are present in the *high-energy*-spectra [53]. The high number of fragments leads to a higher risk of false association to specific peptides in the identification process. In general, data from DIA devices, including MS^E^, leads to an increased complexity in the interpretation of the identification of peptides [64]. The presented approach for feature identification needs a customized database that enables a flexible adaptation to the specific study, e.g., by incorporating reaction-specific modifications, but it is independent from universal databases. The only way to identify a feature’s structure would be an experimental de novo-sequencing of the peptides. However, this would need an even more complex programming approach and a more advanced computational setup, as every amino acid needs to be identified, characterized, and assigned to its position in the peptide [65].

The approach discussed in the present study gives an idea of the possibilities of data evaluation besides common software that is often device-dependent.

### Supplementary Information

Below is the link to the electronic supplementary material.

Supplementary file1 (DOCX 1.19 MB)

## Data Availability

The datasets generated during and/or analyzed during the current study are available from the corresponding author on reasonable request.
