# Supplementary material for: Python workflow for the selection and identification of marker peptides—proof-of-principle study with heated milk
Source: Anal Bioanal Chem. 2024 Apr 12;416(14):3349–60. doi: 10.1007/s00216-024-05286-w (PMC11106092; doi:10.1007/s00216-024-05286-w)
Supplement: Supplementary file 1 — Supplementary file1 (DOCX 1.19 MB) [file 216_2024_5286_MOESM1_ESM.docx]

**Analytical and Bioanalytical Chemistry**

**Electronic Supplementary Material**

**Python Workflow for the Selection and Identification of Marker Peptides – Proof-of-Principle Study with Heated Milk**

Gesine Kuhnen ^a, b; †^, Lisa-Carina Class ^a, c, †^, Svenja Badekow ^a^, Kim Lara Hanisch ^a^, Sascha Rohn ^b^, Jürgen Kuballa ^a,^ *

^a^ GALAB Laboratories GmbH, Am Schleusengraben 7, 21029 Hamburg, Germany

^b^ Department of Food Chemistry and Analysis, Institute of Food Technology and Food Chemistry, Technical University Berlin, Gustav Meyer Allee 25, 13355 Berlin, Germany

^c^ Hamburg School of Food Science, Institute of Food Chemistry, University of Hamburg, Grindelallee 117, 20146 Hamburg, Germany

^†^ Both authors contributed equally to this work.

* Corresponding author: Dr. Jürgen Kuballa, GALAB Laboratories GmbH, Am Schleusengraben 7, 21029 Hamburg, Germany. E-mail address: Juergen.Kuballa@galab.de


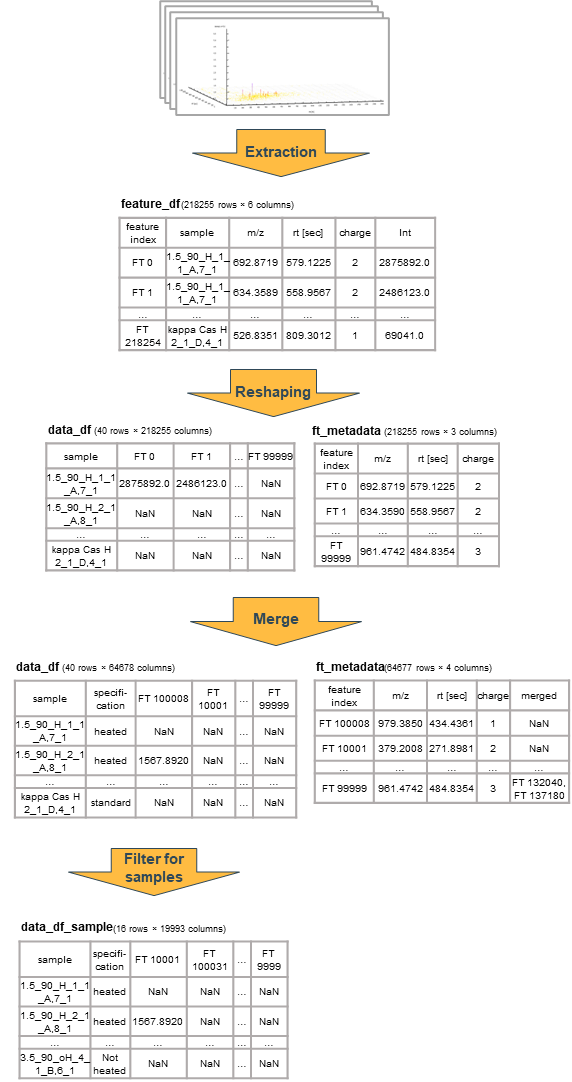


**Fig. S1** Transformation of the data shape during the feature engineering. Firstly, the features are extracted from the mzML-files and saved into a data frame (“Extraction”). The data frame is reshaped into two separate DataFrames (“Reshaping”). Similar features are merged (“Merge”). In the last step the data frame was filtered for the sample data (“Filter for samples”)


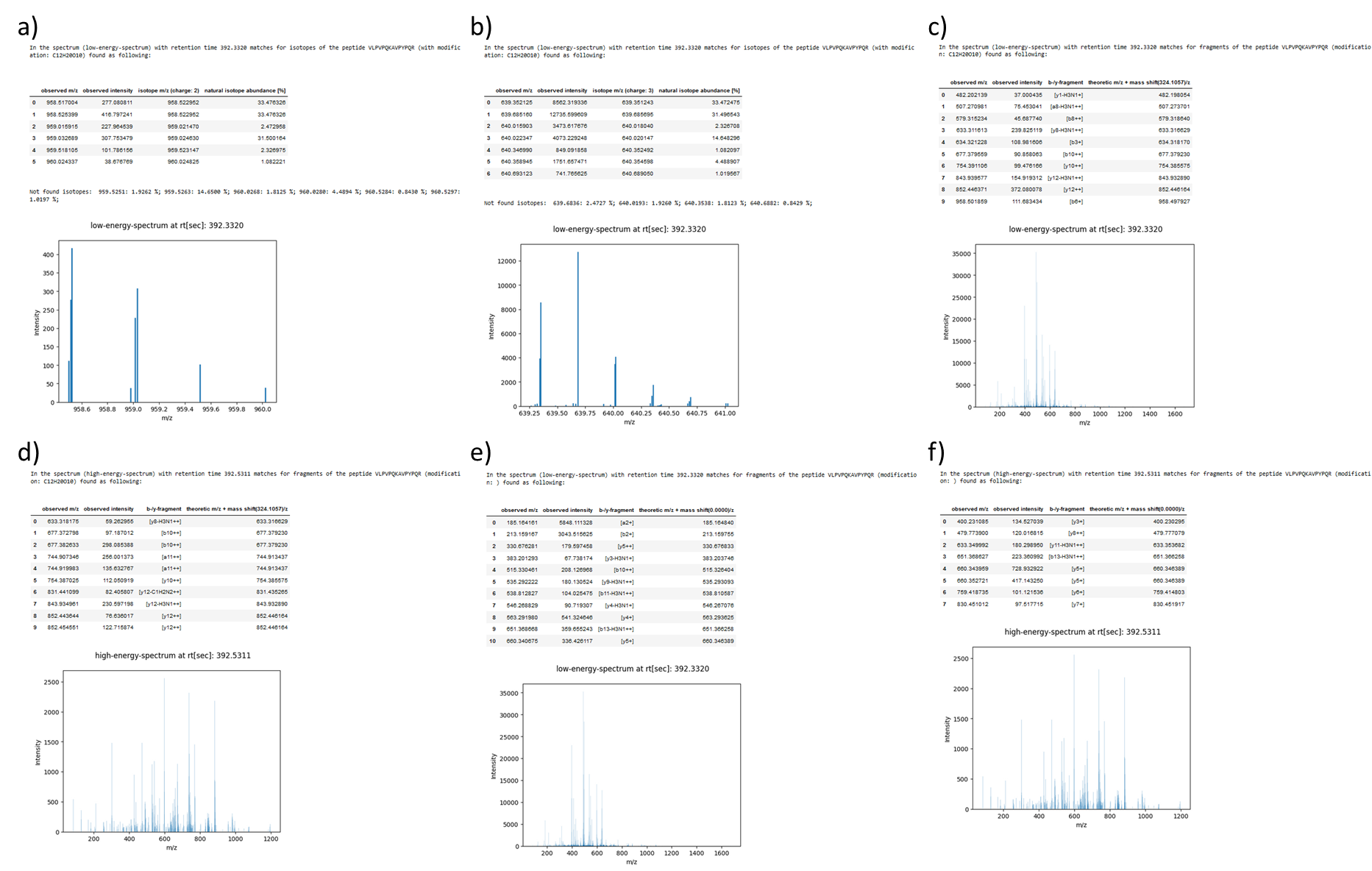


**Fig. S2** Output of the proceeded identification of feature FT43359 as peptide VLPVPQKAVPYPQR with lactulosyllysine as modification. The presented search was proceeded with a heated sample (“1.5_90_H_2_1_A,8_1.mzML“). a) Output of the search for isotopes in the low-energy spectrum (charge: 2). b) Output of the search for isotopes in the low-energy spectrum (charge: 3). c) Output of the search for fragments of the peptide with mass shift due to lactulosyllysine in the low-energy-spectrum. d) Output of the search for fragments of the peptide with mass shift due to lactulosyllysine in the high-energy-spectrum. e) Output of the search for fragments of the peptide without modification in the low-energy-spectrum. f) Output of the search for fragments of the peptide without modification in the high-energy-spectrum


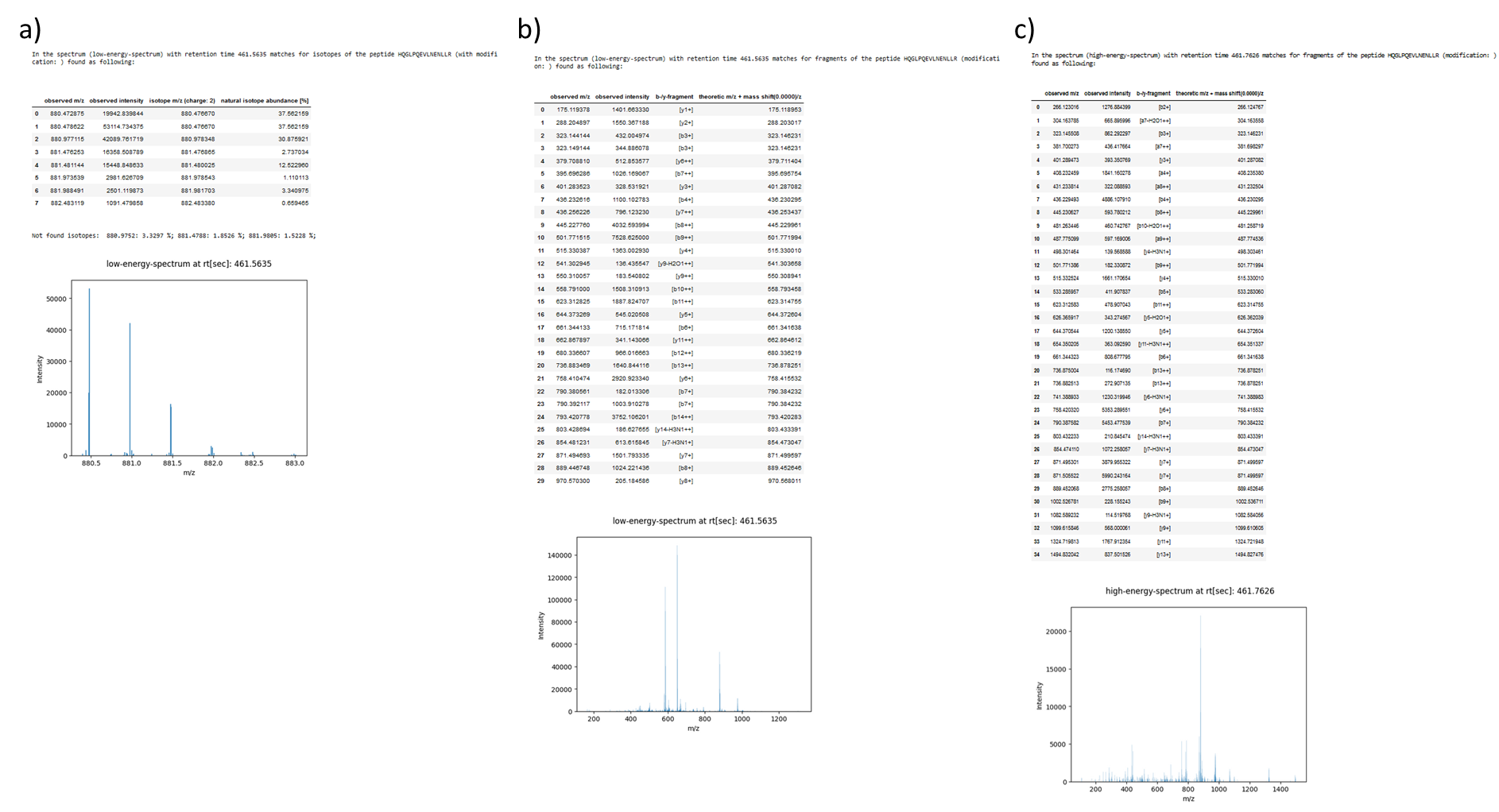


**Fig. S3** Output of the proceeded identification of feature FT48247 as peptide HQGLPQEVLNENLLR. The presented search was proceeded with a heated sample (“1.5_90_H_2_1_A,8_1.mzML“). a) Output of the search for isotopes of the peptide in the low-energy spectrum (charge: 2). For the single charged peptide, no isotopes were found. b) Output of the search for fragments of the peptide in the low-energy-spectrum. c) Output of the search for fragments of the peptide in the high-energy-spectrum


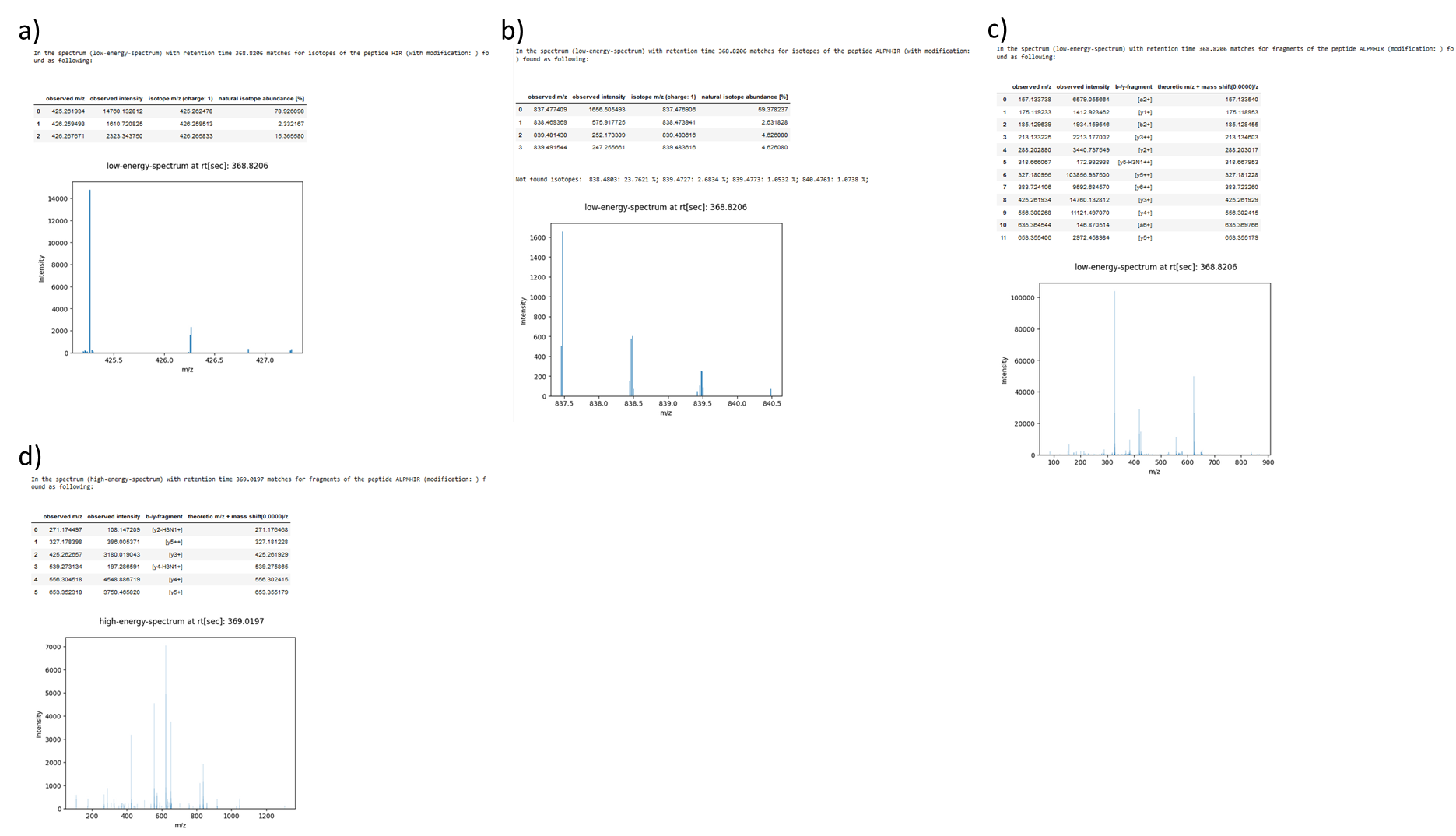


**Fig. S4** Output of the proceeded identification of feature FT11758 as peptide (APLM)HIR. The presented search was proceeded with a heated sample (“1.5_90_H_2_1_A,8_1.mzML“). a) Output of the search for isotopes in the low-energy-spectrum for the peptide HIR, which is equivalent to the y3+ ion of APLMHIR. b) Output of the search for isotopes in the low-energy-spectrum for the peptide APLMHIR. c) Output of the search for fragments of the peptide in the low-energy-spectrum (for “ALPMHIR”). d) Output of the search for fragments of the peptide in the high-energy-spectrum (for “ALPMHIR”)
